# Student perspectives of preparedness characteristics for clinical learning within a fully distributed veterinary teaching model

**DOI:** 10.1371/journal.pone.0249669

**Published:** 2021-05-13

**Authors:** Khalil Saadeh, Joanna B. Aitken, Sharmini Julita Paramasivam, Peter Cockcroft, Kamalan Jeevaratnam

**Affiliations:** 1 School of Veterinary Medicine, Faculty of Health and Medical Sciences, University of Surrey, Guildford, United Kingdom; 2 School of Clinical Medicine, Addenbrooke’s Hospital, University of Cambridge, Cambridge, United Kingdom; University of Macau, MACAO

## Abstract

The transition into the clinical environment is challenging and associated with significant stress and anxiety. This study aimed to examine the perspectives of students on the characteristics important for preparedness for clinical learning and the influence of gender, age, and graduate status on those perspectives. This descriptive, questionnaire-based study of 62 characteristics categorised into six themes was conducted within the Surrey School of Veterinary Medicine completed by 139 students commencing their final clinical year. The Friedman test and post-hoc Wilcoxon signed rank sum test explored for differences in ranking across the themes. Ordinal logistic regression and Mann-Whitney U pairwise comparisons were utilised to investigate for effects of gender, age, and graduate status on theme ranking. There was a significant difference (P <0.05) between medians for themes of preparedness characteristics with comparisons revealing willingness and communication and interaction as the most highly rated characteristics. Knowledge and understanding were viewed as the least important characteristic. Regression and pairwise Mann-Whitney U comparisons confirmed no significant effects (P >0.05) of gender, age or graduate status on student rating of preparedness characteristics. Integrating learning opportunities of those preparedness characteristics in the pre-clinical curriculum may improve students’ preparedness for the clinical environment.

## Introduction

Traditionally, veterinary education is divided into pre-clinical and clinical years. The pre-clinical years, undertaken primarily at university, have been taught on a subject based curriculum model. On the other hand, clinical training predominantly takes place within veterinary teaching hospitals associated with the university. Clinical placements are ultimately considered to be the gold-standard for clinical learning. Placements allow undergraduate students to consolidate, integrate and apply their knowledge in authentic clinical settings, develop clinical skills as well as professional attitudes and behaviours that are required to be a successful clinician. However, the transition from the pre-clinical phase of study into the significantly different clinical learning environment can be challenging and stressful for medical and veterinary students [1] as they can suffer initial clinical anxiety [2]. This is due to a number of factors including differences in learning environments, fear of making mistakes, and work pressures especially regarding examinations and acquiring professional knowledge, skills and attitudes [3, 4].

Progressively, efforts are being made to increase students’ preparedness for the transition to the clinical environment by modifying and adapting the curriculum. Contemporary veterinary curricula have incorporated training in professional skills. These include communication, teamwork, problem solving and business management skills to compliment clinical content [5]. This training aims to increase employability of new graduates and also supports good practice. More recently there has been a shift to a distributed model of clinical training within the curriculum which utilizes a number of clinical sites outside of the veterinary school. This model has been used in other degrees such as medicine, nursing and physiotherapy. However, it is a relatively new concept within UK veterinary education, with the first fully distributed teaching model having been introduced at the University of Surrey in 2014. Within the fully distributed clinical teaching model, veterinary practices are chosen with the intention of providing students with authentic work-place based education which is supportive, organized and provides learning experiences and delivery of high-quality education in their final clinical year of the five year programme. However, limitations with this model related to standardizing the clinical curriculum across the training sites still exist [6]. Nevertheless, a fully distributed model has the advantages of exposing students to a high case-load of primary clinical practice in an authentic environment and context.

Integral to this transition within a fully distributed model, is that students are ready and are prepared for the challenges that they will face in a clinical practice setting. This not only includes background clinical skills and knowledge, but also characteristics such professionalism, interpersonal and communication skills, and the development of emotional resilience. There is a growing recognition and demand from the veterinary profession that undergraduate teaching produces confident, knowledgeable and prepared students that are ready to be exposed to the real world of veterinary practice, and not only survive, but flourish. Fundamental to this is understanding what the students believe are important qualities to acquire when making this transition into clinical learning. Recently, there has been research into supervisor’s perspectives on the characteristics important for preparedness for clinical learning in the disciplines of medicine, pharmacy, nursing, occupational therapy, physiotherapy and speech pathology [4, 7]. Supervisors, particularly in medicine, nursing, and pharmacy, viewed student’s willingness to engage, assist, and practice in the clinical setting as the most important characteristic regarding clinical preparedness [4, 7]. Students’ demonstration of professional skills and behaviours was also ranked very highly [4]. Thus, views on student preparedness valued external professional traits such as professional appearance and willingness to participate more than a specific level of knowledge and understanding. Interestingly, some differences in perspectives existed between disciplines. For example, the importance of willingness and professionalism was ranked higher by nursing than medical supervisors [4]. However, there is little research on preparedness characteristics from a student’s perspective, especially within the veterinary field. Understanding the students’ perspective enables any misalignment to be identified between what is required and what the student perception of preparedness may help us realign preclinical curricula. This is likely to reflect deficits in the pre-clinical curriculum.

Previous work by Chipchase et al. [7] into clinical preparedness identified six themes that can be used as indicators for student preparedness for the clinical learning environment. The six themes are knowledge and understanding, willingness, professionalism, communication and interaction, personal attributes, and professional and interpersonal skills. Therefore, in this study, we explored the students’ perspectives on the characteristics that are important for preparedness for clinical learning. Additionally, we investigate the effects of age, gender and graduate status of students on their perspectives of these characteristics.

## Methods

The aim of the study was to compare students’ perspectives on the characteristics that are important for preparedness for clinical learning. Additionally, it explored the effects of gender, graduate status and age on the ranking of those characteristics by students. Ethical approval for the study was obtained from the University of Surrey’s Ethics Committee (UEC/2018/063/FHMS).

### Study design, setting and participants

This was a descriptive, questionnaire-based study conducted within the School of Veterinary Medicine at the University of Surrey. All students commencing their final clinical year (5^th^ year) in the years of 2018/19 and 2019/20 were invited to participate in the study. Participants provided written informed consent. Questionnaires were distributed and collected within a scheduled session, with approximately 30 minutes given for participants to complete their responses. Overall, 139 responses were collected.

### Questionnaire design

The questionnaire with 62 characteristics, first developed by Chipchase et al. (2012) [7] and used by Banneheke et al. (2017) [4], was modified and adopted for distribution to veterinary students (S1 Appendix). These 62 characteristics were categorised into six themes, namely “Knowledge and Understanding”, “Willingness”, “Professionalism”, “Communication and Interaction”, “Personal attributes”, and “Professional and Interpersonal skills”. Theme headings were removed from the survey to reduce any potential response bias. Item responses were based on a seven-point Likert scale with 1 = not important, 2 = slightly important, 3 = somewhat important, 4 = moderately important, 5 = important, 6 = very important and 7 = extremely important. 0 was provided if the question was not applicable. In addition, a section for demographic data and two open-ended questions for free text comments were included.

### Data analysis

Data were entered into Microsoft Excel (Microsoft Corporation, Redmond, WA, USA). Data presented in this report are expressed as standard box and whiskers plots with central line representing the median, the box representing the interquartile range, the whiskers representing the range between the minimum and maximum values. N refers to the number of individuals in each group. Statistical Package for the Social Sciences (SPSS) Statistics software (IBM, USA) was used to perform the statistical tests. For each participant the mean rank for questions in each theme was calculated. Based on the Shapiro-Wilks normality test results, the non-parametric Friedman test was used to evaluate the hypothesis that the medians of the mean ranks were equal across the themes. Significance for this test is assumed at a P value of < 0.05. The Post-hoc Wilcoxon signed rank sum test was used to explore the statistically significant pairwise comparisons between theme medians. Significance for this test is assumed at a P value of < 0.003 with Bonferroni correction for multiple testing. Ordinal logistic regression was used to examine if age (scale variable), gender (categorical variable) or graduate position (categorical variable) have an effect on the ranking of preparedness characteristics by students. Significance for this test is assumed at a P value of < 0.05. This was confirmed by Mann-Whitney U pairwise comparisons between groups classified based on gender, graduate status and age (categorised into ≤ 24 years, and ≥ 25 years). Significance for this test is assumed at a P value of < 0.008 with Bonferroni correction for multiple testing.

## Results

Across both years surveyed there was a total of 139 responses. Table 1 summarises participant characteristics. There were more females (N = 111; 79.9%) than males (N = 28; 20.1%), more undergraduates (N = 129; 92.8%) than postgraduates (N = 10; 7.2%), and while the age of participants ranged from 21 to 38 there were more participants ≤ 24 years (N = 116; 84.1%; mean = 22.5 years, standard deviation = 0.84) than ≥ 25 years (N = 22; 15.9%; mean = 27.0 years, standard deviation = 2.8).

**Table 1:** Characteristics of student participants.

Characteristic of participants	
**Gender**	N = 139
Female	N = 111 (79.9%)
Male	N = 28 (20.1%)
**Graduate status**	N = 139
Undergraduate	N = 129 (92.8%)
Postgraduate	N = 10 (7.2%)
**Age**	N = 138
≤ 24 years	N = 116 (84.1%)
≥ 25 years	N = 22 (15.9%)

Number (N =) and percentage (%)

### Comparisons between themes

**Fig 1:**
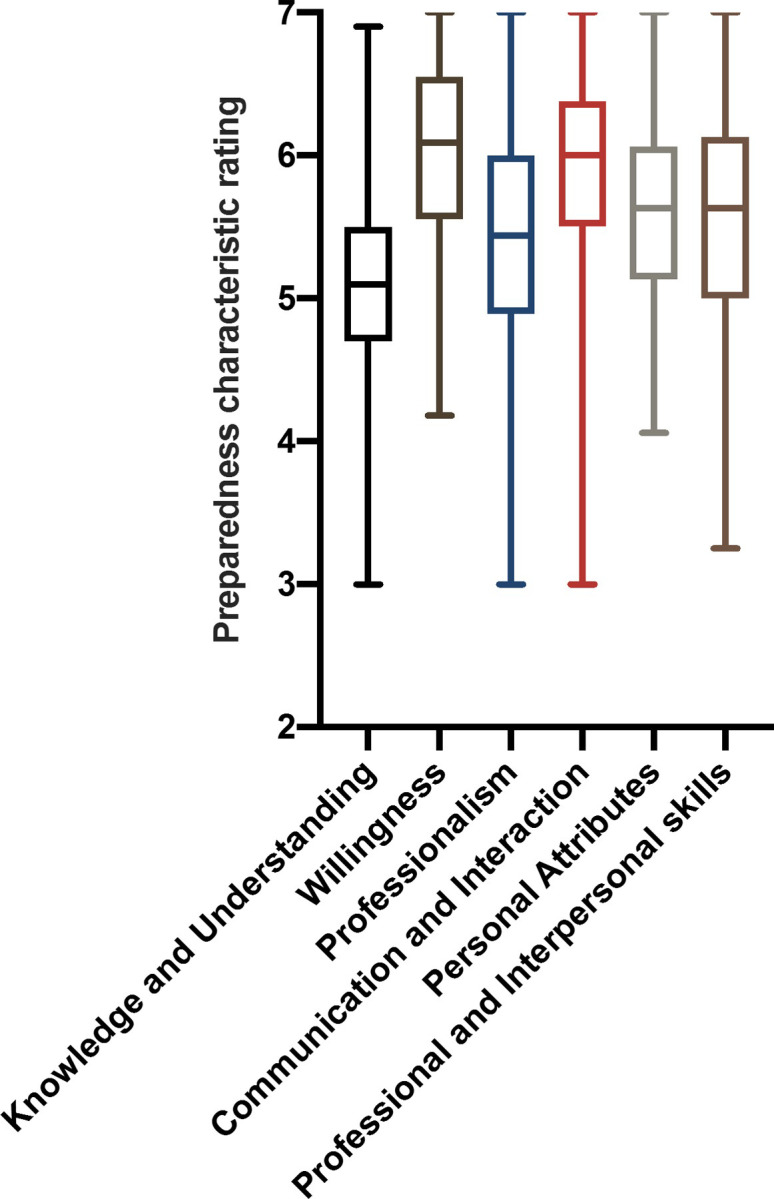
Box and Whiskers plots of students’ responses to preparedness characteristics themes. Median scores for all themes of preparedness for clinical learning were above 5 indicating that they were perceived by students to be important or very important.

Fig 1 shows box and whiskers plots for the scores of the six themes of preparedness for clinical learning. Median scores for all themes of preparedness for clinical learning were above 5 indicating that they were perceived by students to be important or very important. The median rating of the total sample was highest for the theme of willingness (6.09), followed by communication and interaction (6.00), personal attributes (5.63) and professional and interpersonal skills (5.63), professionalism (5.44), and knowledge and understanding (5.10). The Friedman test comparing the medians of the mean ranks across the themes resulted in a statistically significant result (P < 0.05) thus demonstrating that the themes were not perceived to be equal by students. Post-hoc Wilcoxon signed rank sum test confirmed the presence of statistically different (P < 0.003) pairwise comparisons between the groups. Table 2 demonstrates which groups were statistically significantly different from each other.

**Table 2:** Pairwise comparisons in students’ ratings of preparedness characteristics.

Themes compared		Significance
Knowledge and understanding	Willingness	↑; * (P < 0.003)
	Professionalism	↑; * (P < 0.003)
	Communication and interaction	↑; * (P < 0.003)
	Personal attributes	↑; * (P < 0.003)
	Professional and interpersonal skills	↑; * (P < 0.003)
Willingness	Professionalism	↓; * (P < 0.003)
	Communication and interaction	↓;—(P = 0.026)
	Personal attributes	↓; * (P < 0.003)
	Professional and interpersonal skills	↓; * (P < 0.003)
Professionalism	Communication and interaction	↑; * (P < 0.003)
	Personal attributes	↑; * (P < 0.003)
	Professional and interpersonal skills	↑;—(P = 0.006)
Communication and interaction	Personal attributes	↓; * (P < 0.003)
	Professional and interpersonal skills	↓; * (P < 0.003)
Personal attributes	Professional and interpersonal skills	↓;—(P = 0.848)

(↑) indicates a higher median (column 2) than the comparator (column 1), (↓) indicates a lower median (column 2) than the comparator (column 1), (*) indicates statistically significant comparison (P < 0.003), (-) indicates non-statically significant comparison (P > 0.003)

### Student ratings based on gender

Table 3 summarises the median values for the six themes by groups according to gender. Ordinal logistic regression revealed no significant results for the impact of gender on scores of Knowledge and Understanding (P = 0.39), Willingness (P = 0.837), Professionalism (P = 0.381), Communication and Interaction (P = 0.304), Personal attributes (P = 0.602), or Professional and Interpersonal skills (P = 0.423) themes related to preparedness for clinical learning. Thus, demonstrating that gender does not affect students’ perception of the importance of characteristics related to preparedness for clinical learning.

**Table 3:** Students’ ratings (median value) for each preparedness characteristic theme.

Student characteristic		Knowledge and understanding	Willingness	Professionalism	Communication and interaction	Personal attributes	Professional and interpersonal skills
Gender	Female	5.10	6.09	5.44	6.00	5.56	5.63
	Male	5.30	6.14	5.78	5.88	5.69	5.75
Graduate status	Undergraduate	5.10	6.09	5.44	6.00	5.63	5.63
	Postgraduate	5.30	6.23	5.28	6.00	5.47	5.51
Age	≤ 24 years	5.10	6.18	5.44	6.07	5.63	5.63
	≥ 25 years	5.30	6.05	5.56	5.88	5.47	5.57

No significant (P > 0.05) pairwise comparisons were found

The results of the ordinal logistic regression were further confirmed by the lack of significant Mann-Whitney U pairwise comparisons between male and female participants for rankings of Knowledge and Understanding (P = 0.43), Willingness (P = 0.829), Professionalism (P = 0.391), Communication and Interaction (P = 0.317), Personal attributes (P = 0.665), or Professional and Interpersonal skills (P = 0.439) themes. Therefore, confirming the absence of effects of gender on ranking scores of themes related to preparedness for clinical learning.

### Student ratings based on graduate status

Table 3 summarises the median values for the six themes by groups according to graduate status. Ordinal logistic regression revealed no significant results for the impact of graduate status on scores of Knowledge and Understanding (P = 0.718), Willingness (P = 0.533), Professionalism (P = 0.9), Communication and Interaction (P = 0.745), Personal attributes (P = 0.931), or Professional and Interpersonal skills (P = 0.592) themes related to preparedness for clinical learning. Thus, demonstrating that graduate status does not affect students’ perception of the importance of characteristics related to preparedness for clinical learning.

The results of the ordinal logistic regression were further confirmed by the lack of significant Mann-Whitney U pairwise comparisons between undergraduate and postgraduate participants for rankings of Knowledge and Understanding (P = 0.445), Willingness (P = 0.571), Professionalism (P = 0.788), Communication and Interaction (P = 0.375), Personal attributes (P = 0.276), or Professional and Interpersonal skills (P = 0.292) themes. Therefore, confirming the absence of effects of graduate status on ranking scores of themes related to preparedness for clinical learning.

### Student ratings based on age

Table 3 summarises the median values for the six themes by groups according to age. Ordinal logistic regression revealed no significant results for the impact of age on scores of Knowledge and Understanding (P = 0.722), Willingness (P = 0.655), Professionalism (P = 0.541), Communication and Interaction (P = 0.072), Personal attributes (P = 0.22), or Professional and Interpersonal skills (P = 0.714) themes related to preparedness for clinical learning. Thus, demonstrating that age does not affect students’ perception of the importance of characteristics related to preparedness for clinical learning.

The results of the ordinal logistic regression were further confirmed by the lack of significant Mann-Whitney U pairwise comparisons between ≤ 24 and ≥ 25 participants for rankings of Knowledge and Understanding (P = 0.321), Willingness (P = 0.673), Professionalism (P = 0.771), Communication and Interaction (P = 0.116), Personal attributes (P = 0.114), or Professional and Interpersonal skills (P = 0.481) themes. Therefore, confirming the absence of effects of age on ranking scores of themes related to preparedness for clinical learning.

### Responses to open-ended questions

Students were invited to comment on attributes they believed were also important to clinical learning but had not been covered in the questionnaire. Thirty-nine students (28.1%) responded to these open questions. The salient suggestions made were matched with the themes in the questionnaire and summarised in Table 4. Overall, the answers incorporated characteristics relating to personal attributes, willingness, communication and interaction, and professional and interpersonal skills. Students’ resilience was the most common suggestion accounting for 15% of the answers.

**Table 4:** Summary of categorised responses by students to open comments regarding important preparedness characteristics.

Theme	Suggested characteristic	Frequency
Personal attributes	Resilience	7
Personal attributes	Confidence	6
Personal attributes	Adaptability: to new places, social settings, and examination styles	4
Personal attributes	Humility and able to help with menial tasks (e.g. cleaning)	4
Personal attributes	Sociability	2
Personal attributes	Passion and desire to learn	2
Personal attributes	Care and empathy	2
Personal attributes	Humour	2
Personal attributes	Perseverance	2
Personal attributes	Integrity	1
Personal attributes	Stress management	1
Personal attributes	Able to take initiative	1
Personal attributes	Enthusiastic	1
Willingness	Willingness to accept criticism and improve	4
Communication and interaction	Knowing when it is appropriate to ask questions	1
Communication and interaction	Professional communication	1
Communication and interaction	Professional use of social media	1
Professional and interpersonal skills	Being able to decipher and handle significant amounts of data/information	1
	Knowing how and where to find help/support if needed	4

## Discussion

The present study investigated the perceptions of students of veterinary medicine entering the final year of clinical training regarding characteristics important for the preparedness of clinical training. Additionally, it explored the effects of gender, age and graduate status on the perception of the six examined themes. A knowledge of students’ perceptions regarding characteristics for the preparedness of clinical training is important for adapting teaching in the pre-clinical years towards students’ requirements producing a cohort better equipped at handling the challenging transition to clinical learning through which the undergraduate students transforms into a work-ready clinician.

The 139 participants that took part in the study represent a unique cohort of students that are of the first in the United Kingdom to enter a fully distributed model of clinical veterinary education. The original questionnaire has been previously used to examine perceptions of clinical supervisors in other health professions such as medicine and nursing [4, 7]. The questionnaire assesses characteristics which are appropriate for a range of healthcare-based professionals, making the findings of our study applicable for a variety of different health professions including veterinary and human medical schools.

Across multiple health professions, including nursing, veterinary and human medicine, the transition from the familiar environment of pre-clinical education to the unfamiliar setting of clinical learning can be challenging and stressful and as such may be associated with initial significant anxiety and failure to effectively utilise learning opportunities [1, 2, 8, 9]. Thus, students report a feeling of unpreparedness for clinical practice and liken the experience to being thrown in the deep [1, 10, 11]. This highlights an important need to adapt the pre-clinical teaching curricula of health professions such as veterinary medicine so as to better prepare students for this transition into clinical practice. The findings of the present study investigated students’ perceptions of the characteristics that prepare them for this transition and demonstrated that all six themes (knowledge and understanding, willingness, professionalism, communication and interaction, personal attributes, and professional and interpersonal skills) had a median score over 5 indicating that they were all perceived by students to be important or very important in preparing them for clinical learning. This means that the much-needed comprehensive adjustments to the pre-clinical curriculum to increase students’ preparedness for clinical learning should involve learning outcomes that target all of the six themes.

The results revealed that not all characteristics were rated equally with significant differences between the medians of the groups. The themes of willingness (6.09) and communication and interaction (6.00) were perceived by students to be the most important preparedness characteristics for clinical learning. Willingness denoted characteristics relevant to students’ willingness to engage, assist, learn and practise, whereas communication and interaction denoted characteristics relevant to students’ demonstration of communication and interactive ability [7]. These were followed by personal attributes (5.63) and professional and interpersonal skills (5.63), and professionalism (5.44). Hence, knowledge and understanding (5.10) was rated as the least important of those characteristics. Our findings of veterinary students complement previous studies of perceptions of clinical supervisor’s in other health professions. When supervisors, rather than students, in the fields of occupational therapy, physiotherapy, speech pathology [7] as well as supervisors from medicine, pharmacy and nursing [4] were surveyed they ranked willingness and professionalism then personal attributes as the most important characteristics. Thus, this emphasises the importance of willingness as the most important characteristic for clinical learning as viewed by both students and supervisors and across the different health professions. However, differences do arise with regards to the relative importance of communication compared to professionalism. Veterinary students viewed communication and interaction as more important whereas clinical supervisors ranked professionalism higher. This may reflect a difference in attitudes between students and supervisors regarding which characteristics are important for preparedness for clinical learning. Professionalism, denoting characteristics relevant to students’ demonstration of professional skills and behaviours, is based on essentials of clinical competencies, ethics and legal understanding [4, 12]. Supervisors have greater experience of such skills and as a result might value professionalism more than students. Importantly, however, this difference in attitude between supervisors and students may be reflected in measures taken to prepare students for clinical learning. In this regard, it is essential to remember to take into account students’ views, not just those of supervisors, as to what allows students to effectively prepare and engage in their clinical learning and consider curriculum changes accordingly.

Students and supervisors across different health professions agreed that knowledge and understanding, relevant to students’ demonstrating knowledge and understanding of related theory, processes and tasks, was the least important characteristic. This is arguably a reasonable position as knowledge can be taught and acquired in the course of clinical training and does not represent the primary a barrier to student engagement with the clinical environment [9, 13].

Gender differences in attitudes and barriers in clinical practise have been reported in a number of health professions [14–17]. For example, female veterinary students demonstrated higher levels of emotional empathy and care towards animals than their male peers [15, 16]. The present findings found no significant effects of gender on ranking of preparedness characteristics. Hence, no significant differences between male and female groups were found. Thus, demonstrating that gender does not affect students’ perception of the importance of characteristics related to preparedness for clinical learning.

In some countries, such as the United States, the veterinary and human medicine courses are graduate entry courses only. In the UK, these courses primarily admit undergraduate students, but graduate entry is also available for mature students. The difference in age and hence maturity and life experiences can have significant effects on student characteristics such as confidence and professionalism. Previous reports have suggested mature students had a better transition into clinical life than their undergraduate peers [18]. They were able to draw on their previous experiences, feel less confused, more confident and demonstrate better performance [18–21]. Thus, we explored the effects of age and graduate status on perceptions of characteristics important for clinical learning. The present study found no significant effects of age or graduate status on ranking of preparedness characteristics. Hence, no significant differences between ≤ 24 and ≥ 25groups or between undergraduate and postgraduate groups were found. Thus, demonstrating that neither age nor graduate status affect students’ perception of the importance of characteristics related to preparedness for clinical learning.

These findings demonstrate considerable consistency regarding perceptions of preparedness characteristics finding that these perceptions are not altered by students’ gender, age or graduate status. This allows measures targeting these characteristics to be implemented across the whole cohort with expectations of equal efficacy in increasing preparedness to clinical learning across these different groups.

The open comments by students offered a valuable insight into the characteristics important for student clinical preparedness. Those descriptions were mapped to the clinical preparedness themes. Those predominantly related to personal attributes but also included willingness, communication and interaction, and professional and interpersonal skills. Interestingly, some of these suggestions related to aspects not surveyed in the questionnaires. For example, the ability to seek support during the transition to clinical learning was mentioned multiple times. This highlights the importance of providing information about and access to a range of support services including supervisors and pastoral care when located in a practice setting especially during the challenging period of transition.

In this study, the scores obtained for each theme identify the priority areas from the students’ perspectives and accordingly should be used by curriculum planners to incorporate into the pre-clinical curriculum sessions targeting the development and emphasis of these prioritised attributes. It is important that students are made aware of these six themes from the beginning of their course so that they get maximal opportunity to learn and demonstrate these characteristics. Curriculum planners should provide teaching sessions and assessments measuring these themes especially willingness, communication and interaction and affirmative personal attributes. For example, the multiple mini interview used for selection of students in certain health professions have been shown to be effective in assessing non-cognitive attributes, such as professionalism, motivation, interest, empathy, and integrity [22–25]. Furthermore, it has been suggested that the introduction of an orientation course prior to the clinical phase where these characteristics are emphasised would assist in reducing the stress of transition [4].

### Limitations and future directions

The number of respondents to the survey was significant (n = 139) allowing for valuable findings to be made regarding students’ perspectives on the characteristics important for preparedness for clinical learning. Importantly, the sample size was comparable to previous studies in the field [4, 7]. Nonetheless, future studies should perform power calculations. Subdividing responses by demographic variables produced group with smaller unequal sample sizes. This in fact reflected demographic distribution in the Surrey School of Veterinary Medicine (e.g. significantly more undergraduate entry than postgraduate entry students). Future studies should aim to achieve higher responses from underrepresented demographic groups, particularly postgraduate ≥ 25 years male students. This will increase power and allow us to more confidently generalise the present findings.

Furthermore, future studies should investigate additional factors that may influence students’ perspective of the relative importance of preparedness characteristics. For example, whether a student’s aptitude and performance on the veterinary course relates to particular perspectives on preparedness characteristics. This may be done by utilising a student’s ranking at the end of their pre-clinical years correlates to favouring certain characteristics. It is not unlikely that more academically inclined students may rank “Knowledge and Understanding” higher than “Communication and Interaction”. Other factors may include involvement in student life and leadership positions. Moreover, a deeper analysis of external professional traits and internal factors (e.g. academic knowledge) may assist in accounting for the variability in responses reflected in the data range around observed medians (see Fig 1).

## Supporting information

S1 Appendix Student perspective. (DOC)
