# Supplementary material for: Student perspectives of preparedness characteristics for clinical learning within a fully distributed veterinary teaching model
Source: PLoS One. 2021 May 13;16(5):e0249669. doi: 10.1371/journal.pone.0249669 (PMC8118455; doi:10.1371/journal.pone.0249669)
Supplement: S1 Appendix — (DOC) [file pone.0249669.s001.doc]

**STUDENT PERSPECTIVE**

Research title: Student and clinical supervisors’ perspective on student preparedness for clinical learning in veterinary medicine

Your responses to this questionnaire will be used only for research purposes to help us determine the key factors influencing student preparedness for clinical learning. Your responses will be kept **CONFIDENTIAL.**

Thank you for your cooperation.

**SECTION A. Personal Information (Please answer this section)**

**Please respond to the following statements by writing the appropriate answer in the space provided or ticking (🗸) in the space next to the options.**

Name (optional): Age in present year:

Ethnicity:

Gender: Male Female Non-specified

Undergraduate 🞏 Postgraduate 🞏 Cohort/Year of expected graduation ________________________

**SECTION B**

**Please respond to each statement below that best reflects how you feel about the importance of these factors for your preparedness for clinical learning when you enter clinical training from the pre-clinical phase.**

**Please circle *ONE* most appropriate response for each statement. Please answer every item.**

| **No** | **Statements** | **Rating** | | | | | | | |
| --- | --- | --- | --- | --- | --- | --- | --- | --- | --- |
|  |  | Not applicable | Not important | Slightly important | Somewhat important | Moderately important | Important | Very important | Extremely important |
| 1 | I can demonstrate sound theoretical knowledge in basic sciences | 0 | 1 | 2 | 3 | 4 | 5 | 6 | 7 |
| 2 | I can demonstrate a thorough knowledge of therapy practices relevant to the area | 0 | 1 | 2 | 3 | 4 | 5 | 6 | 7 |
| 3 | I know how to access information when a gap in knowledge or need for further information is identified | 0 | 1 | 2 | 3 | 4 | 5 | 6 | 7 |
| 4 | I can demonstrate basic knowledge of the key features of common conditions | 0 | 1 | 2 | 3 | 4 | 5 | 6 | 7 |
| 5 | I can demonstrate some understanding about the department or organization where I will be undertaking the placement | 0 | 1 | 2 | 3 | 4 | 5 | 6 | 7 |
| 6 | I can demonstrate knowledge of basic treatment principles for common conditions | 0 | 1 | 2 | 3 | 4 | 5 | 6 | 7 |
| 7 | I can demonstrate knowledge of forms of treatment that may be detrimental to a patient | 0 | 1 | 2 | 3 | 4 | 5 | 6 | 7 |
| 8 | I can demonstrate knowledge of other professions and their roles | 0 | 1 | 2 | 3 | 4 | 5 | 6 | 7 |
| 9 | I have an understanding of my own learning style | 0 | 1 | 2 | 3 | 4 | 5 | 6 | 7 |

| **No** | **Statements** | **Rating** | | | | | | | |
| --- | --- | --- | --- | --- | --- | --- | --- | --- | --- |
|  |  | Not applicable | Not important | Slightly important | Somewhat important | Moderately important | Important | Very important | Extremely important |
| 10 | I can demonstrate knowledge of the clinical assessment tools my educator is using to assess me | 0 | 1 | 2 | 3 | 4 | 5 | 6 | 7 |
| 11 | I can demonstrate reasoning skills | 0 | 1 | 2 | 3 | 4 | 5 | 6 | 7 |
| 12 | I am willing to work as a team with peers, colleagues and other health professionals | 0 | 1 | 2 | 3 | 4 | 5 | 6 | 7 |
| 13 | I am willing to ask questions and clarify to ensure understanding | 0 | 1 | 2 | 3 | 4 | 5 | 6 | 7 |
| 14 | I am willing to try new techniques | 0 | 1 | 2 | 3 | 4 | 5 | 6 | 7 |
| 15 | I am willing to discuss and exchange ideas to maximize patient care | 0 | 1 | 2 | 3 | 4 | 5 | 6 | 7 |
| 16 | I am willing to receive feedback/constructive criticisms | 0 | 1 | 2 | 3 | 4 | 5 | 6 | 7 |
| 17 | I have willingness to take on board any appropriate requested task | 0 | 1 | 2 | 3 | 4 | 5 | 6 | 7 |
| 18 | I am willing to stray from my comfort zone | 0 | 1 | 2 | 3 | 4 | 5 | 6 | 7 |
| 19 | I am willing to adhere to positive workplace culture and routines e.g. tidying up, cleaning | 0 | 1 | 2 | 3 | 4 | 5 | 6 | 7 |
| 20 | I am willing to take responsibility for my own learning | 0 | 1 | 2 | 3 | 4 | 5 | 6 | 7 |
| 21 | I am willing to self-evaluate | 0 | 1 | 2 | 3 | 4 | 5 | 6 | 7 |
| 22 | I have a thorough understanding of the code of conduct and ethics for my profession | 0 | 1 | 2 | 3 | 4 | 5 | 6 | 7 |
| 23 | I understand my role and I am able to verbalize this | 0 | 1 | 2 | 3 | 4 | 5 | 6 | 7 |
| 24 | I arrive at the placement on time | 0 | 1 | 2 | 3 | 4 | 5 | 6 | 7 |

| **No** | **Statements** | **Rating** | | | | | | | |
| --- | --- | --- | --- | --- | --- | --- | --- | --- | --- |
|  |  | Not applicable | Not important | Slightly important | Somewhat important | Moderately important | Important | Very important | Extremely important |
| 25 | I dress appropriately for the workplace and placement (e.g. closed in shoes, uniform if appropriate, visible ID badge, hair, fingernails, jewellery) | 0 | 1 | 2 | 3 | 4 | 5 | 6 | 7 |
| 26 | I comply with professional matters such as confidentiality | 0 | 1 | 2 | 3 | 4 | 5 | 6 | 7 |
| 27 | I attend each day having demonstrated appropriate follow up from previous day | 0 | 1 | 2 | 3 | 4 | 5 | 6 | 7 |
| 28 | I make appropriate contact with facility/educator prior to the placement commencing | 0 | 1 | 2 | 3 | 4 | 5 | 6 | 7 |
| 29 | I am prepared for the first day having completed the appropriate pre-reading and bringing learning resources relevant for the clinical area(s) | 0 | 1 | 2 | 3 | 4 | 5 | 6 | 7 |
| 30 | I display ability to maintain professional boundaries with patients/clients | 0 | 1 | 2 | 3 | 4 | 5 | 6 | 7 |
| 31 | I respectfully engage with people from a wide range of cultures and backgrounds | 0 | 1 | 2 | 3 | 4 | 5 | 6 | 7 |
| 32 | I can demonstrate effective communication and interpersonal skills verbal, non-verbal and listening) with clients across the lifespan | 0 | 1 | 2 | 3 | 4 | 5 | 6 | 7 |
| 33 | I am able to liaise with key stakeholders, such as organizing appointments | 0 | 1 | 2 | 3 | 4 | 5 | 6 | 7 |
| 34 | I am able to communicate professionally with members of the multidisciplinary team | 0 | 1 | 2 | 3 | 4 | 5 | 6 | 7 |

| **No** | **Statements** | **Rating** | | | | | | | | |
| --- | --- | --- | --- | --- | --- | --- | --- | --- | --- | --- |
|  |  | Not applicable | | Not important | Slightly important | Somewhat important | Moderately important | Important | Very important | Extremely important |
| 35 | I can demonstrate respectful and non-judgmental communication | 0 | | 1 | 2 | 3 | 4 | 5 | 6 | 7 |
| 36 | I have the capacity to adjust my interaction style to meet the needs of the audience, whether it be colleagues, clients or others | 0 | | 1 | 2 | 3 | 4 | 5 | 6 | 7 |
| 37 | I can demonstrate effective written communication skills, in charts, letters and information for clients | 0 | | 1 | 2 | 3 | 4 | 5 | 6 | 7 |
| 38 | I can demonstrate enthusiasm and interest in the placement | 0 | | 1 | 2 | 3 | 4 | 5 | 6 | 7 |
| 39 | I show initiative | 0 | | 1 | 2 | 3 | 4 | 5 | 6 | 7 |
| 40 | I am sensitive/empathetic to patient’s needs and concerns | 0 | | 1 | 2 | 3 | 4 | 5 | 6 | 7 |
| 41 | I have the ability to manage stress levels | 0 | | 1 | 2 | 3 | 4 | 5 | 6 | 7 |
| 42 | I can demonstrate a desire to learn | 0 | | 1 | 2 | 3 | 4 | 5 | 6 | 7 |
| 43 | I can demonstrate the ability to self-reflect on performance, interactions and outcomes | 0 | | 1 | 2 | 3 | 4 | 5 | 6 | 7 |
| 44 | I have self-awareness of own limitations and I am honest about current level of knowledge & skills | 0 | | 1 | 2 | 3 | 4 | 5 | 6 | 7 |
| 45 | I can demonstrate the ability to apply myself | 0 | | 1 | 2 | 3 | 4 | 5 | 6 | 7 |
| 46 | I am attentive | 0 | | 1 | 2 | 3 | 4 | 5 | 6 | 7 |
| 47 | I am curious and ask questions | 0 | | 1 | 2 | 3 | 4 | 5 | 6 | 7 |
| 48 | I am proactive | 0 | | 1 | 2 | 3 | 4 | 5 | 6 | 7 |
| 49 | I am diligent | 0 | | 1 | 2 | 3 | 4 | 5 | 6 | 7 |
| 50 | I am self-directed | 0 | 1 | | 2 | 3 | 4 | 5 | 6 | 7 |

| **No** | **Statements** | **Rating** | | | | | | | |
| --- | --- | --- | --- | --- | --- | --- | --- | --- | --- |
|  |  | Not applicable | Not important | Slightly important | Somewhat important | Moderately important | Important | Very important | Extremely important |
| 51 | I am helpful | 0 | 1 | 2 | 3 | 4 | 5 | 6 | 7 |
| 52 | I am polite | 0 | 1 | 2 | 3 | 4 | 5 | 6 | 7 |
| 53 | I am creative | 0 | 1 | 2 | 3 | 4 | 5 | 6 | 7 |
| 54 | I am assertive | 0 | 1 | 2 | 3 | 4 | 5 | 6 | 7 |
| 55 | I can demonstrate time management skills e.g. use of a diary, to do lists | 0 | 1 | 2 | 3 | 4 | 5 | 6 | 7 |
| 56 | I can demonstrate organizational skills | 0 | 1 | 2 | 3 | 4 | 5 | 6 | 7 |
| 57 | I have good verbal and written skills | 0 | 1 | 2 | 3 | 4 | 5 | 6 | 7 |
| 58 | I can demonstrate good observational skills | 0 | 1 | 2 | 3 | 4 | 5 | 6 | 7 |
| 59 | I have research skills to find basic information to fill in existing knowledge gaps | 0 | 1 | 2 | 3 | 4 | 5 | 6 | 7 |
| 60 | I have foundation skills for the area of practice | 0 | 1 | 2 | 3 | 4 | 5 | 6 | 7 |
| 61 | I can demonstrate social skills e.g. the ability to relate personably | 0 | 1 | 2 | 3 | 4 | 5 | 6 | 7 |
| 62 | I can demonstrate problem-solving skills | 0 | 1 | 2 | 3 | 4 | 5 | 6 | 7 |

Are there any other student attributes that you believe are important for clinical learning?

…………………………………………………………………………………………………………………………………………………………….

…………………………………………………………………………………………………………………………………………………………….

Your comments/ suggestions

…………………………………………………………………………………………………………………………………………………………….

…………………………………………………………………………………………………………………………………………………………….
